# Complete mitogenome of the Common Koel *Eudynamys scolopaceus* Linnaeus 1758 (Aves: Cuculidae)

**DOI:** 10.1080/23802359.2022.2073846

**Published:** 2022-05-10

**Authors:** Guohai Wang, Chuangbin Tang, Jinlan Li, Qiuchan Huang, Jialin Nong, Lijuan Wei, Qihai Zhou

**Affiliations:** a College of Chemistry and Bioengineering, Guangxi Normal University for Nationalities, Chongzuo, China; b Personnel Department of Guangxi Normal University for Nationalities, Chongzuo, China; c College of Mathematics, Physics and Electronic Information Engineering, Guangxi Normal University for Nationalities, Chongzuo, China; d Key Laboratory of Ecology of Rare and Endangered Species and Environmental Protection, Ministry of Education, Guangxi Normal University, Guilin, China

**Keywords:** Common Koel, mitochondrial genome, Cuculidae, phylogeny

## Abstract

The Common Koel *Eudynamys scolopaceus* Linnaeus 1758, belongs to the family Cuculidae and order Cuculiformes. This omnivorous bird exhibits obligate nest parasitism. Here, the complete mitogenome of *E. scolopaceus* was determined and phylogenetically compared with those of other Cuculidae species. The newly sequenced complete mitogenome was a circular DNA molecule with a size of 17,610 bp (OM115963). This mitogenome had a higher A + T content (57.58%) than G + C content (42.42%). Phylogenetic analysis revealed that *E. scolopaceus* was most closely related to *Eudynamys taitensis* and the genus *Cuculus*, providing useful molecular information for further research on the phylogeny of the family Cuculidae.

Determining the mitochondrial genome sequences of birds facilitate research on their genetic evolution and genetic relationships(Slack et al. 2006; Kriegs et al. 2007; Wang et al. 2007; Sun, Liu, and Lu 2020; Sun, Liu, Min, et al. 2020; Sun et al. 2021). The Common Koel *Eudynamys scolopaceus* Linnaeus 1758, belongs to the family Cuculidae and order Cuculiformes (Srivastava et al. 2012). This omnivorous bird exhibits obligate nest parasitism. Both male and female Common Koels are medium-sized. The male is black and shows light blue brightness, although the brightness of the lower body is not significant. The upper body of the female is dark brown with a slight metallic green reflection. The head is dark brown in color and covered by white spots. These birds often exhibit longitudinal lines on the head which turn into transverse spots on the tail and flying feathers. The iris is red, the mouth is light green, and the feed is gray. Loud and clear kow-wow sounds are emitted when calling; the call is distinctive and highly recognizable. These birds often use the nests of Common Magpie *Pica pica* Linnaeus,1758, Black-Collared Starling *Gracupica nigricollis* Paykull,1807, and Red-Billed Blue Magpie *Urocissa erythroryncha* Boddaert, 1783 to lay their eggs (Singh et al. 2015; Sun et al. 2018). We sequenced the mitochondrial DNA of *E. scolopaceus* and analyzed its phylogenetic and evolutionary relationships in Cuculiformes and Cuculidae.

This study was reviewed and approved by the Animal Ethics Committee of Guangxi Normal University for Nationalities. The samples were collected and stored at the Institute of Chemistry and Bioengineering, Guangxi Normal University for Nationalities, No. 23 Fozi Road, Jiangzhou District, Chongzuo City, Guangxi Province (107°23′28.97′′E, 22°23′12.41′′N) in March 2021 (http://www.gxnun.edu.cn/, Guohai Wang, 1016729581@qq.com), voucher number ZJ-2106. Genomic DNA was extracted using an Ezup column animal genome DNA extraction kit (B518251-0100; Sangon Biotech, Shanghai, China), according to the manufacturer’s protocol. Sequencing and sequence assembly were performed as previously described (Sun et al. 2021; Wang et al. 2021).

The mitogenome is 17,610 bp in length (GenBank accession no. OM115963), and its nucleotide composition is 33.04% A, 30.10% C, 12.33% G, and 23.53% T, with a higher A + T (57.58%) than G + C (42.42%) content. Nine overlapping regions were identified. Our sequence was similar to *Eudynamys taitensis*, which contains one control region and 37 genes (2 rRNA genes, 13 protein-coding genes, and 22 tRNA genes). Among these 37 genes, 28 are encoded by the heavy strand, while nine are encoded by the light strand. All 13 protein-coding genes started with ATN (ATA and ATG) codons, except for *COX1*, which started with GTG. Typical stop codons (TAG and TAA) were observed in most protein-coding genes, except for the incomplete terminal codons T for *COX3* and *ND4*, AGA for *ND1*, and AGG for *COX1*. The BLAST comparison with the recently published mitogenome of the Common Koel (OL639163) showed sort by query coverage of 98% and percent identity of 96.63%.

We determined the phylogenetic relationships between *E. scolopaceus* and 11 other Cuculiformes based on 13 protein-coding genes. *Phylloscopus schwarzi* was selected as the outgroup. A phylogenetic tree was constructed using the maximum-likelihood method using IQ-TREE v1.6.8 under the edge-linked partition model for 5000 standard bootstraps. Phylogenetic analysis showed that *E. scolopaceus* belongs to the family Cuculidae and that species in the other family within the order Cuculiformes clustered well in one group (Figure 1). The phylogenetic trees showed that the target species *E. scolopaceus* is closely related to *E. taitensis* and the genus *Cuculus*. Species in the family Cuculidae require further comprehensive analysis.

**Figure 1.**
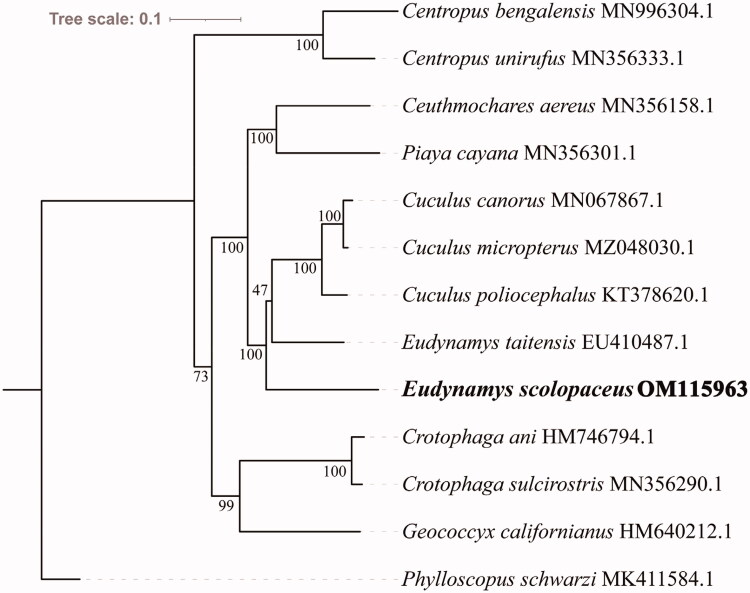
Maximum-likelihood tree of 12 Cuculiformes species and one outgroup based on 13 protein-coding genes. The number at each node is the bootstrap probability. The GenBank accession numbers are listed after the species names. The *E. scolopaceus* mitogenome is marked in bold font.

## Data Availability

The data that support the findings of this study are available in the NCBI GenBank database at https://www.ncbi.nlm.nih.gov/, reference number OM115963. The associated BioProject, BioSample, and SRA numbers are PRJNA743997, SAMN20064762, SRR15046853, respectively (https://www.ncbi.nlm.nih.gov/sra/?term=SRR15046853).
